# 

**Published:** 1872-04

**Authors:** 


### CONTENTS:

FOREIGN CORRESPONDENCE.............. 193

SELECTIONS:

The Process of Decomposition........ 197
Clinical Lecture on Infantile Paralysis, etc.................................. 205
The Cæsarean Operation in the U. S.. 210
On Dyspepsia of Liquids............. 212
Paper on Cancrum Oris.............. 213
A Case of Bright's Disease, etc....... 220
Cerebro-Spinal Meningitis, or "Spotted Fever,"........................ 226

SELECTIONS—*continued.*

Malingering Extraordinary .......... 232
Enuresis .............................. 233
The Plague of 1871 in Buenos Ayres.. 235

EDITORS' BOOK TABLE................. 238

EDITORIAL............................ 239

ITEMS, NEWS AND GOSSIP.............. 240

LOOT ................................. 249

